# Orbital learning: a novel, actively orchestrated decentralised learning for healthcare

**DOI:** 10.1038/s41598-024-60915-9

**Published:** 2024-05-07

**Authors:** Neeraj Kavan Chakshu, Perumal Nithiarasu

**Affiliations:** Zienkiewicz Institute for Modelling, Data and AI, Bay Campus, Fabian Way, Crymlyn Burrows, Swansea University, Swansea, Wales SA1 8EN UK

**Keywords:** Decentralised learning, Digital health, Data security, Data privacy, Mathematics and computing, Computational science, Health care

## Abstract

A novel collaborative and continual learning across a network of decentralised healthcare units, avoiding identifiable data-sharing capacity, is proposed. Currently available methodologies, such as federated learning and swarm learning, have demonstrated decentralised learning. However, the majority of them face shortcomings that affect their performance and accuracy. These shortcomings include a non-uniform rate of data accumulation, non-uniform patient demographics, biased human labelling, and erroneous or malicious training data. A novel method to reduce such shortcomings is proposed in the present work through selective grouping and displacing of actors in a network of many entities for intra-group sharing of learning with inter-group accessibility. The proposed system, known as Orbital Learning, incorporates various features from split learning and ensemble learning for a robust and secure performance of supervised models. A digital embodiment of the information quality and flow within a decentralised network, this platform also acts as a digital twin of healthcare network. An example of ECG classification for arrhythmia with 6 clients is used to analyse its performance and is compared against federated learning. In this example, four separate experiments are conducted with varied configurations, such as varied age demographics and clients with data tampering. The results obtained show an average area under receiver operating characteristic curve (AUROC) of 0.819 (95% CI 0.784–0.853) for orbital learning whereas 0.714 (95% CI 0.692–0.736) for federated learning. This result shows an increase in overall performance and establishes that the proposed system can address the majority of the issues faced by existing decentralised learning methodologies. Further, a scalability demo conducted establishes the versatility and scalability of this platform in handling state-of-the-art large language models.

## Introduction

The digitalisation of healthcare for autonomous patient care in various healthcare systems is curtailed by arduous frameworks for data sharing^1,2^. Sharing of patient data amongst healthcare units, social-care units and research facilities is quintessential for dynamic training, testing and deployment of diagnostic or prognostic deep learning algorithms. However, sharing data mandates ethical, technical and legal scrutiny^3–5^ and associated challenges.

A large number of governments and healthcare institutions are investing in curating local patient data and training individual deep learning algorithms^6,7^. These algorithms, however, having been predominantly trained on the local dataset, have their peak performance limited and biased to a testing or local environment^8,9^. When exposed to external data, they have been observed to show drastically reduced performance, thereby, generalisability of local models for universal adoption is hindered^8^.

**Figure 1:**
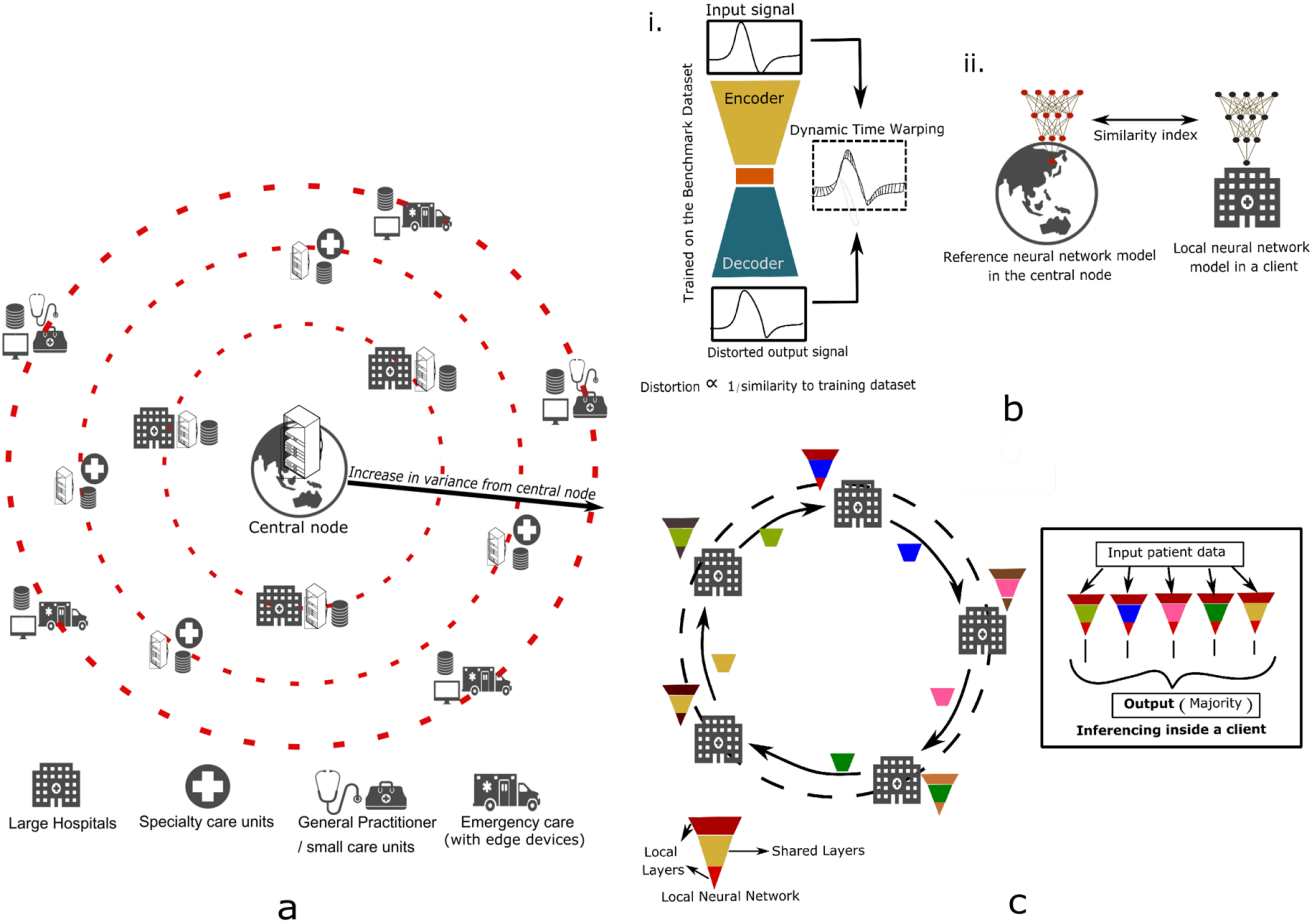
(**a**) Orbital Learning (OL), selectively grouped healthcare clients on a decentralised network to be locally collaborative but globally accessible learning. (**b**) Two types of assessors that establish the performance of a client. (i) Auto-encoder neural network that helps determine clients’ data similarity to the the benchmark dataset. (ii) Similarity index, Spearman’s rank-order correlation in our work, to establish similarity of clients model with the reference model in the central node. (**c**) The functioning of orbital system taking place within an orbit and the bagging ensemble method that takes place during inferencing within a client in the orbit.

To enhance AI algorithms beyond local data without sharing data between participants (healthcare units), decentralised learning over a network of participants is seen as a possibility^10^. In such a network, local data is held privately by every participant of the network. However, the locally trained intelligence is aggregated and shared amongst them. Such a system allows for data protection and at the same time provides a collaborative approach to comprehensive intelligence.

## Related work

Though various network topologies have been proposed, two popular decentralised systems, federated learning^11–16^ and swarm learning^17^, are being tested in healthcare^18–20^. The primary difference between the two is that the former uses a centralised server for intelligence aggregation, whereas the latter employs a peer-to-peer system for the same purpose.

Decentralised learning faces several challenges in design and data-sharing, as highlighted in recent studies^21–23^. These challenges include the drop in performance due to the aggregating algorithm or data poisoning by a participant, which may result from erroneous data generation, tampering, or malicious activity. Additionally, accommodating participants with varying computational capacities is challenging, and heterogeneity in data quality and quantity affects overall performance, such as Non-Independent Identical Distribution (*Non-IID*), data biases, and temporal imbalance. Model inversion attacks may also compromise data privacy by reverse-engineering the shared intelligence to recreate the training data.

To address these challenges, various modified forms of decentralised learning have been proposed. Split federated learning^24^ shares only some layers of a deep learning model to prevent model inversion attacks. Tiered Federated Learning^25^ groups participants based on their training time into temporal tiers, while Generative Adversarial Networks-based federated learning^26^ generates synthetic data to address data imbalance issues.

An emerging concern within decentralised networks is the vulnerability to prompt insertion attacks. These attacks involve the malicious injection of deceptive prompts or commands that can mislead or derail the functioning of the network. Such vulnerabilities are particularly concerning as they can compromise the integrity and reliability of shared intelligence, potentially leading to incorrect or biased outcomes. Addressing the challenge of prompt insertion attacks is crucial for the future development of secure and robust decentralised learning systems. As of now, comprehensive solutions to this specific problem remain underdeveloped, marking it as an important area for future research and innovation.

However, these solutions address individual drawbacks, and there is currently no comprehensive system capable of addressing all challenges within a single architecture. We offer a novel system to conduct decentralised learning, based on active grouping and displacement of participants. This system, known as the Orbital Learning (OL), utilises novel methods along with various concepts adopted from existing decentralised methods. Fundamentally, the proposed system is designed to group participants on an intelligence-sharing network into different orbits based on how their local data quality and rate affect the overall shared intelligence, without any access to their data. The proposed system enables displacement of any participants to a different orbit. Intelligence sharing takes place independently within each of these groups (orbits) but can be accessed by other groups when required. In what follows, we study this system’s functioning on a publicly available ECG-dataset, compare its performance against an existing decentralised learning system, describe its setup and mechanisms.

## Orbital learning setup

Decentralised learning on OL platform comprise of fundamental functional elements (see Fig. 1a). *Clients* are the participants on the intelligence sharing network who house their local patient data, train their neural network models, and share intelligence. The central node is designated to orchestrate the training, testing, movement, and deployment of neural network models, and can either be hosted independently or within one of the larger clients. The benchmark patient dataset is a reference patient database made available to all clients on the network, which is used for training a reference neural network model for benchmarking and calibrating clients’ performance. The central node uses this benchmark dataset to train a reference Neural Network (NN) model which forms the central referential point for orchestrating client movement in the system. Orbits represent groups of clients with similar data and temporal homogeneity, with similar impacts on the shared sections of NN.

To establish similarity amongst the clients, two fundamental characteristics are analysed: Impact on shared intelligence and melioration rate. Impact on shared intelligence is determined by two sub-characteristics: input data and labelling qualities. The former is affected by demographics, patterns, and the quality of local patient data, while the latter is affected by the quality of patient care provided to the client based on the available expertise. These characteristics are measured by *Assessors*, algorithms designed to benchmark local data against the benchmark dataset and referential neural network within the client, thereby avoiding any data sharing. Melioration rate is a result of the clients’ training times, which is affected by both its data collection rate and computational speeds during training of the local neural network.

The shared layers, a section of hidden layers in a NN, adapted from split learning^24,27–29^, form the basic mode of sharing intelligence between the clients (see Fig. 1c). An individual copy of the layers is held by each client in the orbit, trained, and transferred with one other client after each melioration. By placing clients in an orbit, shared layers can be transferred from one client to the neighbouring client, either in a clockwise or anticlockwise manner (see Fig. 1c). However, intelligence is not shared but accessible between orbits, and shared layers from a different orbit can be trained upon only if it has been obtained from an inner orbit to avoid creating new biases into inner orbits with comprehensive data, as outer orbits generally have more biased or lower quality data.

## Methods

The OL system has four distinctive steps necessary for its components to function properly. The first step involves assessing the impact of each client on the shared intelligence, without sharing patient data. This is done using two types of assessors: one to analyse the similarity of input patient data of a client with that of the benchmark dataset held by the central node, and the other to assess the overall impact of a client on the shared intelligence. The second step involves initiating any project on the proposed system, where the central node trains the reference NN model on the benchmark patient dataset and transfers a copy of this reference model to every client on the network. After an initial set number of meliorations without any intelligence sharing, each client is assessed for similarity with that of the central node, and clients are grouped based on similarity using K-means clustering. The third step involves continuous functioning of the system beyond initiation, with intelligence being cyclically shared and trained within an orbit by transfer of the local copy of shared layers of every client to one of their neighbours, while also being assessed for impact before intelligence sharing. Any client found to have an impact magnitude beyond a set threshold from that of the orbit’s average values is removed from the orbit and regrouped with clients in another orbit with similar impact on shared intelligence. Finally, the fourth step involves utilising the shared intelligence for actual inferencing of patient data, where a local NN model is used on every client for diagnosing or monitoring, by incorporating the concept of ensemble learning via using several sets of shared layers within the orbit.

### Experimental procedure

Experiments were conducted to benchmark the proposed OL system against other decentralised methods, Federated Learning (FL), Swarm Learning (SL) and SL with split model sharing (SSL). We split the data in each client into 80% training and 20% testing subsets. The testing subset allowed for estimating the impact of shared intelligence on local data inference. In terms of execution, a total of 15 meliorations were carried out by each of the client during the experiment, with first 5 meliorations during initiation and the rest during maintenance. Similarly, 15 federated or peer-to-peer aggregation rounds were carried out on the same clients on the FL platform with weighted federated averaging, and SL platforms for benchmarking the performance.

In addition to the above experiments, an additional demonstration was conducted to analyse the proposed system’s capacity to handle complex models to establish its relevance in handling state-of-the-art natural language processing based models. This demonstration included deployment of a multimodal model capable of handling text and ECG waveform data to generate a text, classifying the diseases as a one line output. This demo is not compared with other methods due to computational and framework limitations.

### Dataset

**Figure 2:**
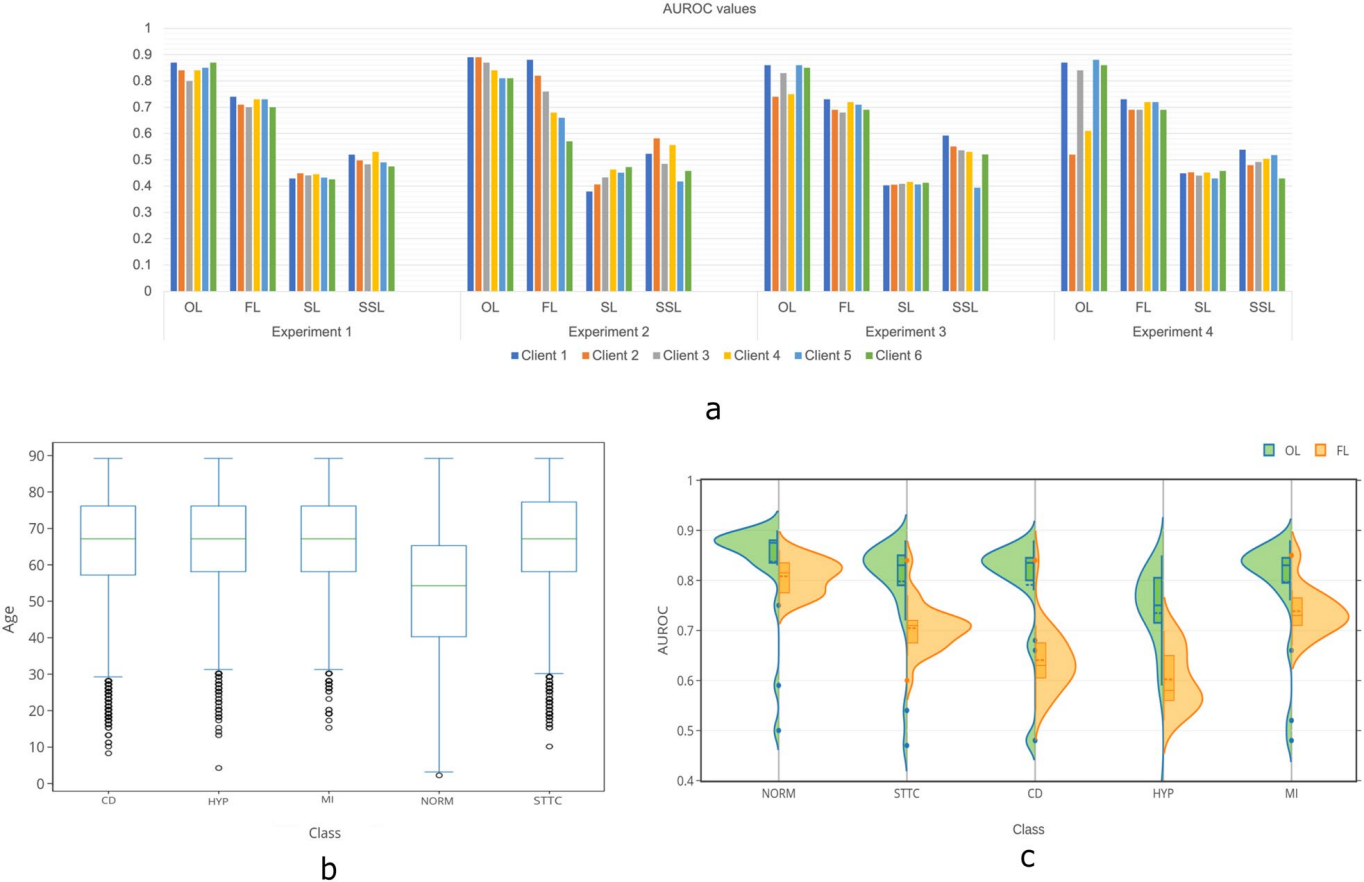
(**a**) Micro-average Area Under Receiving Operating Curve (AUROC) distributions for Orbital Learning (OL), Federated Learning (FL), Swarm Learning (SL) and Swarm Learning with Split model sharing (SSL) obtained for all the four experiments. (**b**) Box plot displaying the distribution of age for each class (label) in the dataset used in the present work. (**c**) Violin plot showing the distribution of AUROC values, distinctively taken, for each class across all clients and all experiments for Orbital Learning vs Federated Learning. Dots mark the outliers in the data.

The patient dataset used in the experiments is taken from the PTB-XL ECG dataset^32–34^. This dataset consists of 21799 clinical 12-lead ECGs from 18,869 patients of 10 s length. This database is publicly available and can be accessed from Physionet^34^. Based on the description provided, the data in this dataset was annotated by up to two cardiologists, who assigned potentially multiple ECG statements to each record. The data has been classified into five superclasses, as described in Table 1 and shown in Fig. 2b,c. The superclasses used are (acronym in brackets) Healthy/Normal ECG (NORM), Changes in the ST- and T-wave (STTC), Conduction Disturbance (CD), Hypertrophy (HYP), Myocardial Infarction (MI).

**Table 1:** Classification of ECGs into five super classes in the PTB-XL dataset and their number of records.

Category	Super classification	Description	Number of records
Class 0	NORM	Normal ECG	9517
Class 1	STTC	ST/T Change	5237
Class 2	CD	Conduction disturbance	4901
Class 3	HYP	Hypertrophy	2649
Class 4	MI	Myocardial infarction	5473

In addition to the PTB-XL ECG dataset, MIMIC-IV Clinical Database^39^, MIMIC-IV deidentified free-text clinical notes , and MIMIC-IV Diagnostic Electrocardiogram Matched﻿ Subset^40^ are used to demonstrate scalability of this platform to handle complex multimodal models, that include natural language data. The 4000 admitted entries were extracted with each sample having clinical notes, 12-lead ECG and corresponding disease classification, i.e., International Classification of Diseases (ICD).

### Neural network model

The experiments are carried out using one-dimensional convolutional neural networks (CNN). The ECG data is used to train a CNN model with 10 hidden layers, consisting of an architecture described in the Appendix (Supplementary Fig. S5). For Federated Learning (FL) models, the same CNN model and client data distribution were adopted within Tensorflow-federated architecture^35^. Tensorflow-federated is an open-source library widely adopted for collaborative learning by various publications across domains^36,37^. Further, for simulating Swarm Learning (SL) and SL with Split Model sharing (SSL), PySyft framework was used. Therefore, the performance of the proposed system has been compared with that of the Tensorflow-federated and PySyft frameworks to benchmark the performance and establish the differences and novelties. The following parameters were adopted for the FL model to match those adopted for the proposed Orbital Learning: client optimizer—Adam^38^ with a default learning rate of 0.001, server optimizer—Adam with a default learning rate of 0.001, repeat in clients (local optimization before federated aggregation)—100, and number of federated aggregation rounds—15.

The scalability demo used a multimodal setup of feature extraction of ECG through a pre-trained encoder and then combining it with clinical text for a tokenised input to be passed through a t5-small architecture tasked at predicting all ICDs that can be assigned as a one-line text.

### Impact assessment

For conducting experiments mentioned in this section, we assess the overall impact of a client (without sharing of local data) on the shared intelligence impact through similarity in weights and biases of the client with that of the reference model from the central node (see Fig. 1c). In theory, we can quantify this similarity using a simple correlation index or complex system such as comparative neural networks (ex. discriminators). For this work, we employ Spearman’s rank-order correlation^31^, i.e.,

1 $$\begin{aligned} \rho = 1-\frac{6 \Sigma d_{i}^{2}}{n(n^{2}-1)} \end{aligned}$$

Here, $d_{i}$ is the difference between the two ranks of each observation, *n* is the number of observations, and $\rho$ is Spearman’s rank correlation coefficient. The coefficient values have a range of − 1 to + 1, with + 1 representing the perfect association between the initial trained weights and the locally trained weights of the neural network. The threshold for accepting clients into an orbit was set at $\pm 0.015$ from that of the average orbit value.

The input data quality at each client is measured by comparing it against the benchmark dataset with the help of an auto-encoder neural network. Auto-encoders have the capacity to capture characteristics of a particular dataset and train to represent data in a latent space (see Fig. 1b). Hence, when subjected to input data from a different dataset or dissimilar quality, distorted outputs are produced. A measure between the input and distorted output signals provide a scale of similarity to the benchmark dataset. The Dynamic Time Warping (DTW) algorithm^30^ is used in our work to quantify this measure. DTW measures the similarity between two temporal sequences and provides the distance between them. The values for which varied between 3 and 120 during the experiments. Input signals closer to that of the benchmark dataset (from the initiation phase) had euclidean distance values between 3 and 10, and other signals varied between 40 and 120.

## Results

To analyse the proposed OL system and compare its performance with other decentralised methods of FL, SL and SL with split model sharing (SSL), collectively referred to as other decentralised methods in this section, we conducted four experiments using ECG classifications for arrhythmia. In these experiments, we introduced simulated distributions, tampering of patient data, and label tampering from a publicly available dataset to establish the proposed OL system’s capability in addressing some of the existing drawbacks of decentralised learning. We designed the first two experiments to simulate clients (participants) with *Non-IID* and unbalanced data. We designed the third experiment to simulate erroneous data or data tampering, and the fourth experiment to simulate label tampering or mix-ups. We used a maximum of six clients in these experiments, with each client having 3000 ECG samples.

### Experiment 1: Randomised *Non-IID* and unbalanced data

Orbital learning platform performed effectively in handling *Non-IID* and unbalanced datasets when exposed to such a randomised dataset, based on values shown in Fig. 2a. The ECG dataset being used was randomly grouped into six clients. Further, client distribution was confirmed to have *Non-IID* before commencement of the experiment. The imbalance of samples between classes (labels) within a client was managed through weighted classes during training. As shown in Fig. 2a, OL outperforms other decentralised methods by a significant margin (Fig. 2c). It can also be observed that the variation in AUROC values between the clients is minimal. This experiment was repeated with an additional setting of slow melioration rate (longer time between consecutive meliorations) in Clients 2 and 4 for the 8*th*–10*th* meliorations (see “Related work”). This routine was conducted to determine if our system can handle temporal heterogeneity. The performance of OL did not change as a result of temporal heterogeneity.

### Experiment 2: Age based *Non-IID* and unbalanced data

For demonstrating the capacity of our proposed system in handling demographical heterogeneity, we conducted an experiment where each client had a completely different age group representing an extremely heterogeneous network of clients. The age groups used were 18–39, 40–49, 50–59, 60–69, 70–79, and 80–89. The experiment simulated extreme demographic disparity, a milder form of which can be observed in real-life healthcare settings, especially in large and ageing demographics. We have described the distribution of age for each class (label) in Fig. 2b. Promising results were observed across the clients in this experiment (see Fig. 2a). Our system automatically determined three orbits after initiation, with Clients 1 and 2 in the innermost orbit, Clients 3 and 4 in the second orbit, and Clients 5 and 6 in the outermost orbit. This configuration was maintained autonomously for most of the experiment. A uniform performance is seen with OL, however, the same cannot be said for FL, where the AUROC values vary significantly between 0.57 and 0.88 (see Fig. 2a). Similar lower performances can be seen in SL and SSL. In other words, clients with data containing sharper features dominate the global performance in FL, which, in turn, affects clients’ accuracy unevenly.

### Experiment 3: Data tampering in two clients with random noise

The effect of malicious activity on a decentralised learning network was estimated in this experiment. The primary objective was to evaluate the capability of a decentralised architecture to isolate malicious actors and prevent their activity from having any adverse impact on the shared intelligence. The ECG data from Experiment 1 was tampered in two of the six clients by adding random noise to their local data. Noise was added to Clients 2 and 4 in the range of 0.0–0.2 to a Z-score normalised input data. As shown in Fig. 2a, the OL platform is capable of avoiding any such adverse effects on the overall performance of the network. It automatically isolated Clients 2 and 4, which were subjected to random noise, thereby preventing the performance of other clients from dropping. Some of the outliers in Fig. 2c for OL, are from this experiment and reflect the values seen in Clients 2 and 4. When this setup with malicious actors was put on other decentralised platforms, the performance of every client on the network was brought down. In FL, SL and SSL, malicious clients are not isolated, and the global model is affected, which in turn brings down the performance of the entire network.

### Experiment 4: Label tampering

Experiment 4 was conducted to evaluate the ability of a decentralised system to detect and handle incorrect labelling and data mix-ups, which can directly impact the quality of care provided to patients. The fourth segment in Fig. 2a illustrate the performance of OL and other decentralised methods, when Clients 2 and 4 had their labels shuffled. Similar to Experiment 3, OL was successful in detecting the anomaly in the affected clients and isolating them from the rest of the network. In this case, the two affected Clients remained in two separate orbits throughout the entire experiment. These results demonstrate that the proposed OL system can protect the performance of all clients from erroneous or malicious human labelling and tampering of model weights. Such isolation allows for manual intervention and rectification of the associated problem, followed by the reintroduction of the client to the system. In contrast, when observed in other methods, the affected clients performed normally but reduced the performance of the rest of the clients. As in the case with Experiment 3, some of the outliers in Fig. 2c for OL, are from this experiment and reflect the values seen in Clients 2 and 4.

Detailed AUROC curves for each class within every client is available in the Appendix. Readers are referred to Supplementary Figs. S1, S2, S3, and S4.

### Scalability demo

**Figure 3:**
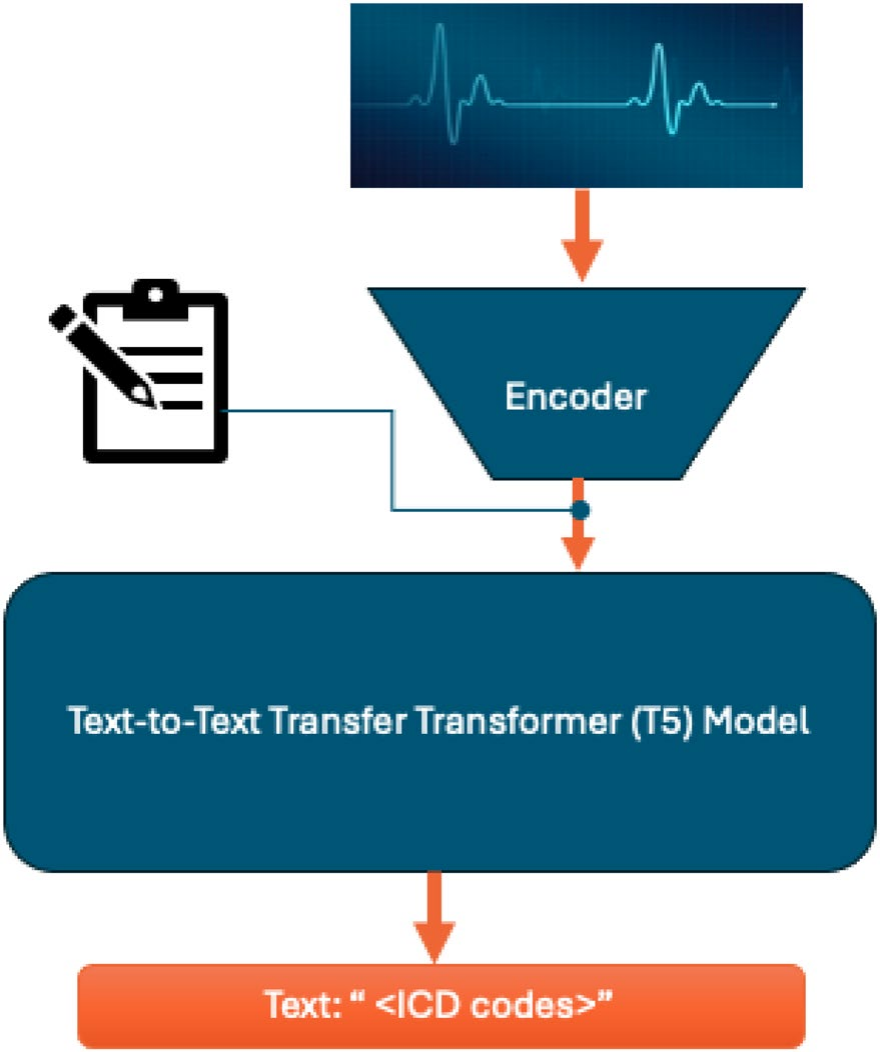
Multimodal input model using Text-to-Text Transfer Transformer (T5) architecture for scalability demo.

This investigation was specifically designed to evaluate the OL’s scalability in supporting complex, multimodal models that utilize advanced language learning mechanisms (LLMs). The primary task for the LLM in this experiment was to analyse and interpret heterogeneous data types, namely textual clinical notes and numerical ECG readings (see Fig. 3). The objective was to seamlessly integrate these modalities to generate concise text outputs that accurately encode the relevant International Classification of Diseases (ICD) codes, corresponding to the diagnosed conditions. This process required the model to not only grasp the semantic nuances of clinical language but also to correlate these narratives with the quantitative patterns found in ECG data, showcasing the model’s ability to handle and synthesize multimodal inputs. The assessors in this demo were the same as experiments before with only ECG analysed among the inputs to establish client’s data similarity to benchmark dataset. In other words, text was not assessed as free-style notes cannot be standardised.

Perplexity scores were employed as a quantitative metric to assess the model’s predictive performance. Perplexity, in this context, measures the model’s ability to predict the next token in the sequence given its current state, with lower scores indicating higher predictive accuracy and a better understanding of the data structure. Scores ranging from 5 to 16 were observed across different test sets, suggesting that the LLM was highly effective at forecasting the sequence of tokens that represent ICD codes, based on the complex interplay of textual and numerical data presented to it. These perplexity values are indicative of the model’s efficiency in navigating through the intricacies of medical documentation and ECG analysis, thus validating the OL platform’s capacity to facilitate sophisticated, AI-powered applications in healthcare analytics. This level of performance underscores the potential of the OL platform as a versatile and powerful tool for advancing medical research and practice, through the integration of cutting-edge computational techniques in the analysis and interpretation of multifaceted health data. The demo had 4 clients with two of them having patients of age only above 60 and the rest having randomly chosen set of patients. The former two are representation of specialised healthcare setting such as geriatric units and the latter two are the representation of general healthcare units. The OL platform placed the clients in two separate orbits. However, it must be noted here that the Spearman’s rank correlation coefficient threshold for being accepted into an orbit was set at $\pm 1.5\times 10^{-5}$ from the orbit’s average value. This was selected through trial and error, while assessing relative sensitivity of this model to different data variations.

## Discussion

Decentralised learning in healthcare, a critical area for the implementation of AI, faces several challenges, notably the presence of *Non-IID* and unbalanced data, temporal heterogeneity, data poisoning, and concerns over data privacy and model security. The OL system proposes innovative solutions to these challenges by structuring participants into orbits based on similarities in data impact and training times, which enhances model performance through targeted ensemble learning and mitigates biases by addressing data quality variability and temporal disparities. This structure also allows for the isolation and correction of data poisoning issues without necessitating the exclusion of participants, thus maintaining the integrity and inclusivity of the learning ecosystem.

Moreover, the OL system is designed with data privacy and security at its core, offering protection against cyber threats such as model inversion attacks, which is paramount in maintaining patient confidentiality. Its flexibility in accommodating varying computational capacities across participants ensures that the system is inclusive and can effectively leverage shared intelligence across different orbits, facilitating transfer learning operations for participants with limited resources.

Incorporating OL within the healthcare sector requires careful navigation of legal and ethical frameworks. Adherence to the GDPR, the UK’s Data Protection Act 2018, and the Medical Devices Regulation (MDR)^41^ is essential for ensuring data privacy and the safety of AI applications in healthcare. Furthermore, the recent EU AI Act^42^ introduces specific obligations for high-risk AI systems, emphasizing the need for transparency, human oversight, and detailed assessments to ensure that AI systems are safe, non-discriminatory, and ethically aligned with societal values.

This comprehensive approach to addressing both technical challenges and regulatory requirements highlights the OL system’s potential to significantly enhance healthcare delivery through decentralised learning. By fostering an environment that is both technologically advanced and ethically grounded, OL promises to drive forward the integration of AI in healthcare, ensuring that it is equitable, secure, and beneficial for all stakeholders involved.

### Supplementary Information

Supplementary Figures.

## Data Availability

Data used in the present work is from the PTB-XL ECG dataset^32–34^. This dataset is publicly available and can be accessed here. MIMIC-IV datasets are publicly available but require credentialed access.

## Code availability

The codes used in this work are in a public repository, and can be found here.
