# Supplementary material for: Orbital learning: a novel, actively orchestrated decentralised learning for healthcare
Source: Sci Rep. 2024 May 7;14:10459. doi: 10.1038/s41598-024-60915-9 (PMC11076556; doi:10.1038/s41598-024-60915-9)
Supplement: Supplementary file 1 — Supplementary Figures. [file 41598_2024_60915_MOESM1_ESM.pdf]

Appendix

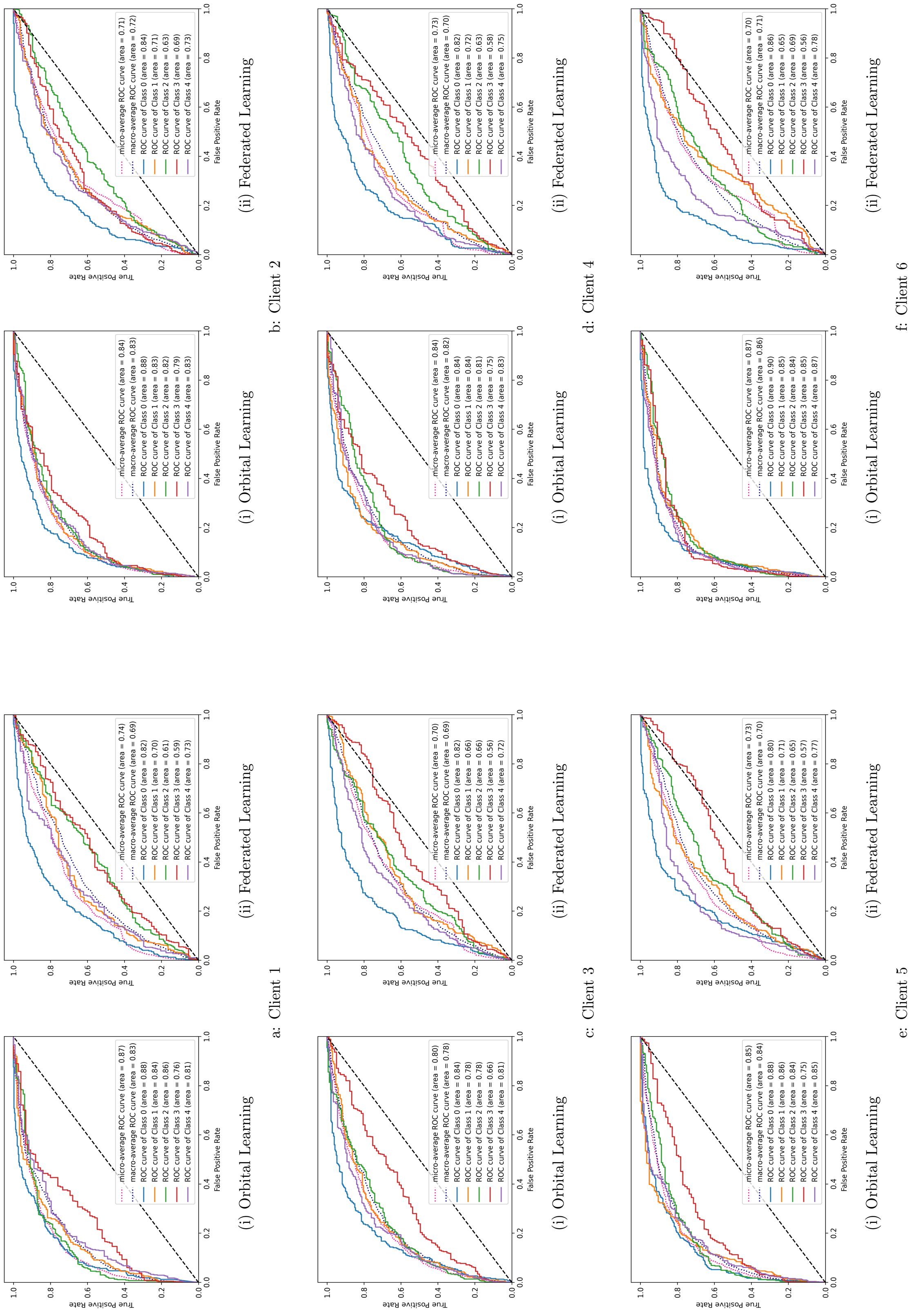

Figure S1: Final receiving operating curve obtained for the six clients for Experiment 1.

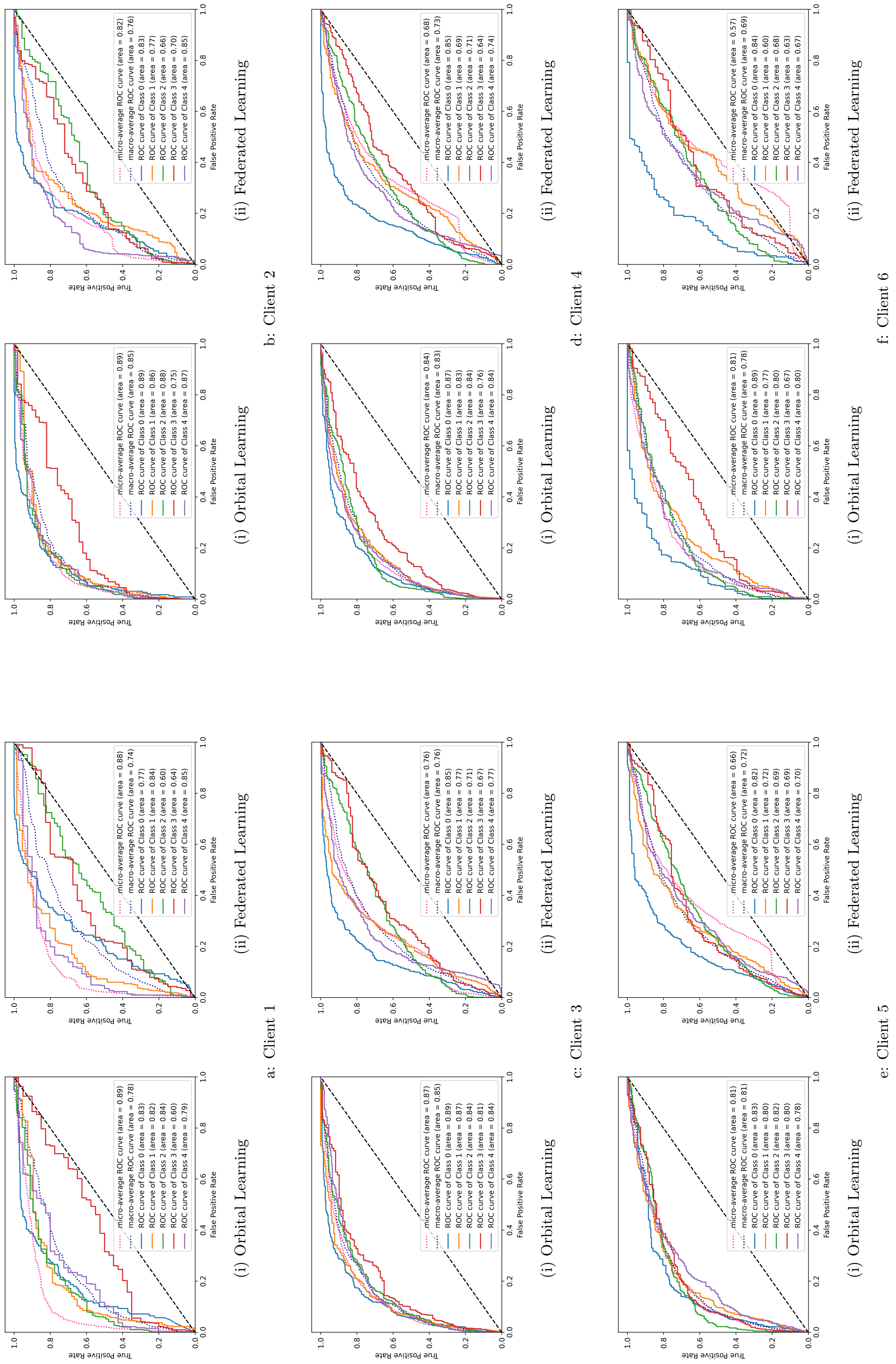

Figure S2: Final receiving operating curve obtained for the six clients for Experiment 2.

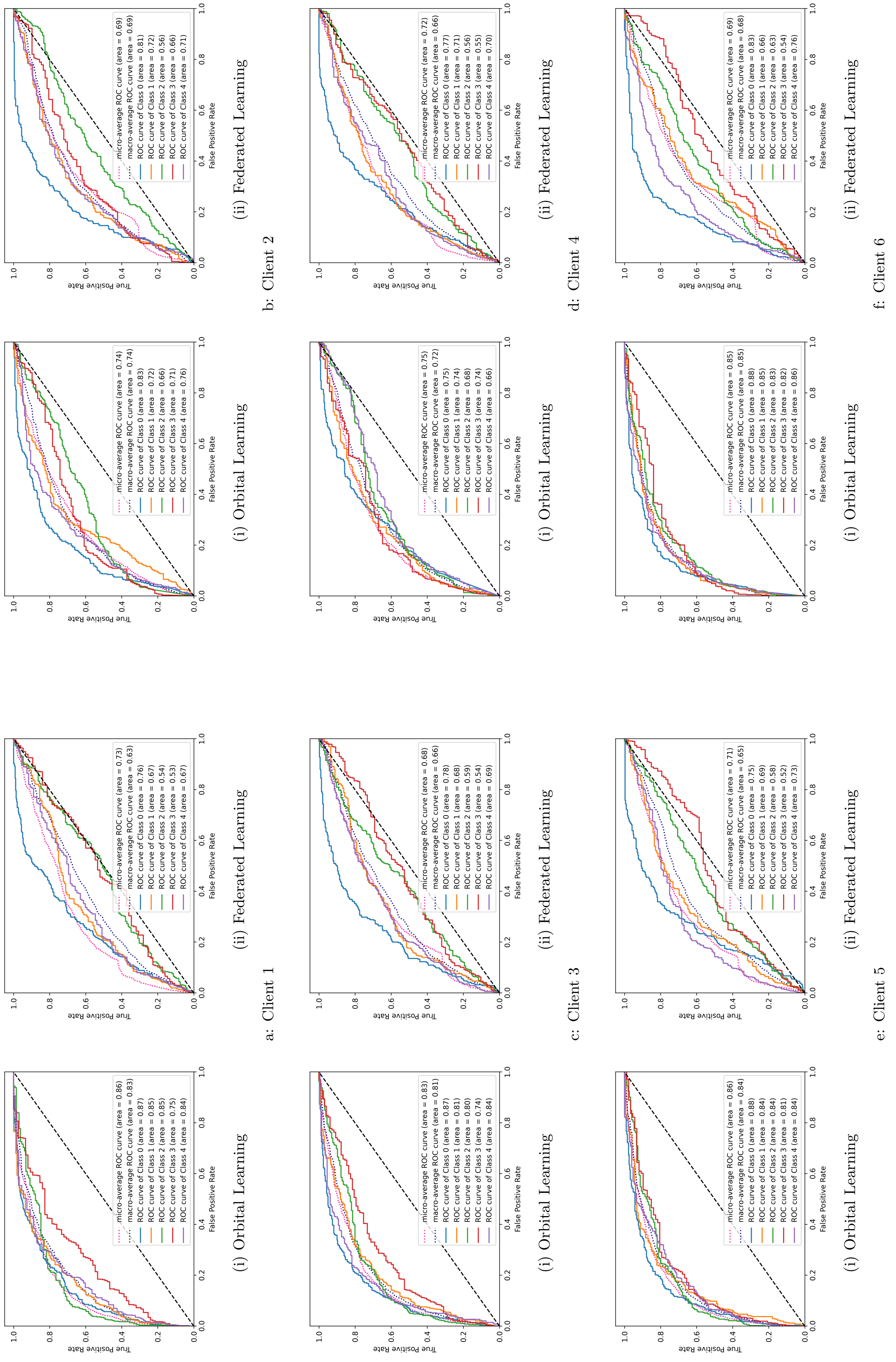

(i) Orbital Learning (ii) Federated Learning
a: Client 1

(i) Orbital Learning (ii) Federated Learning
b: Client 2

(i) Orbital Learning (ii) Federated Learning
c: Client 3

(i) Orbital Learning (ii) Federated Learning
d: Client 4

(i) Orbital Learning (ii) Federated Learning
e: Client 5

(i) Orbital Learning (ii) Federated Learning
f: Client 6

Figure S3: Final receiving operating curve obtained for the six clients for Experiment 3.

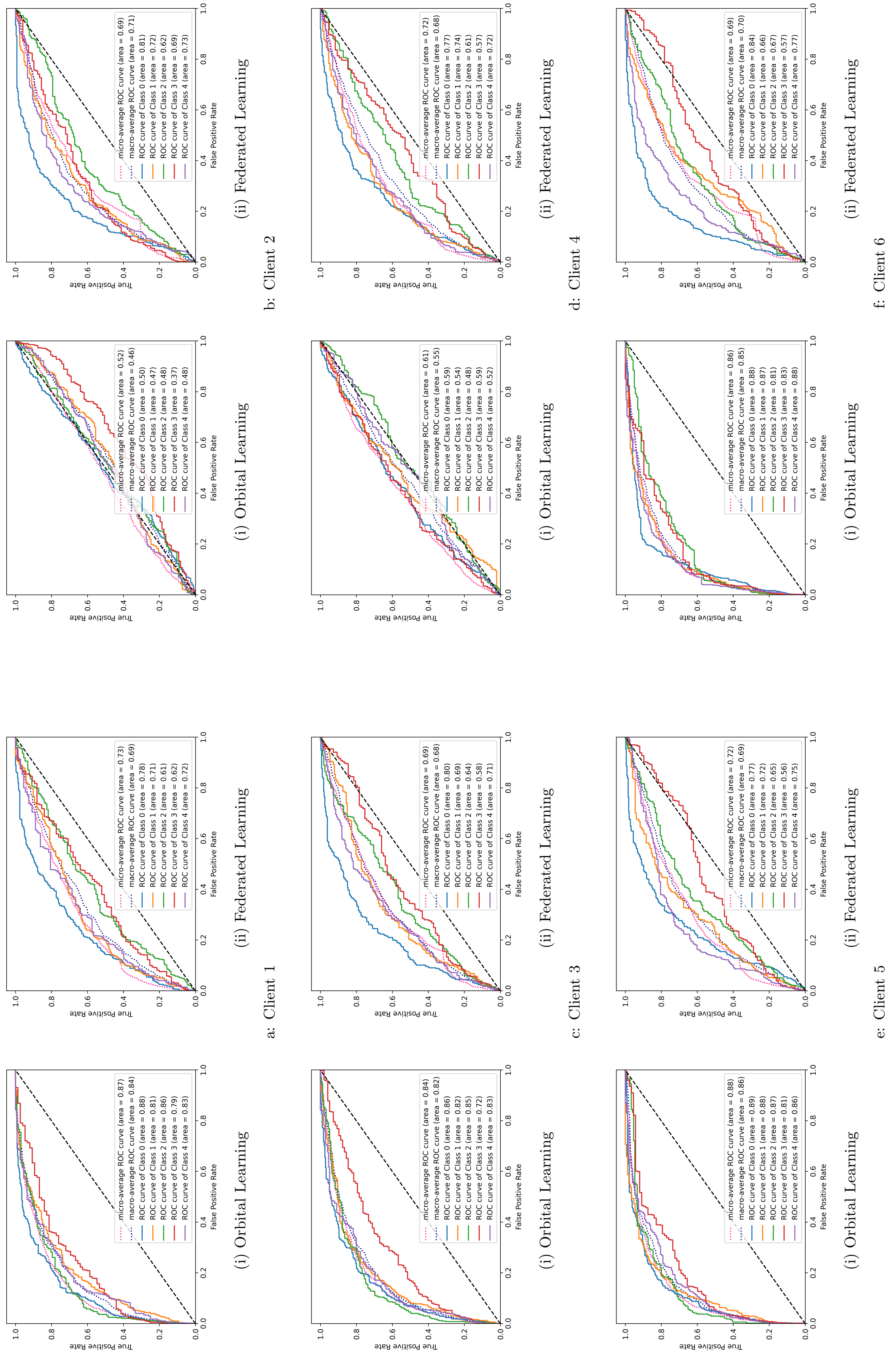

a: Client 1 — (i) Orbital Learning, (ii) Federated Learning

b: Client 2 — (i) Orbital Learning, (ii) Federated Learning

c: Client 3 — (i) Orbital Learning, (ii) Federated Learning

d: Client 4 — (i) Orbital Learning, (ii) Federated Learning

e: Client 5 — (i) Orbital Learning, (ii) Federated Learning

f: Client 6 — (i) Orbital Learning, (ii) Federated Learning

Figure S4: Final receiving operating curve obtained for the six clients for Experiment 4.

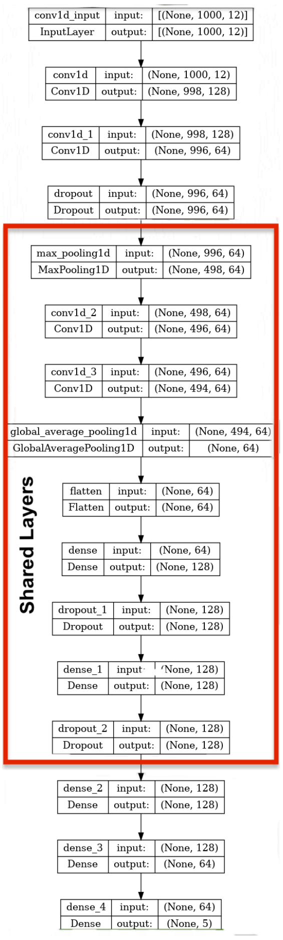

Figure S5: Convolutional Neural network architecture adopted for demonstrating and benchmarking orbital learning with ECG classification for Arrhythmia
